# A model for cofactor use during HIV-1 reverse transcription and nuclear entry^☆^

**DOI:** 10.1016/j.coviro.2013.11.003

**Published:** 2014-02

**Authors:** Laura Hilditch, Greg J Towers

**Affiliations:** University College London, Medical Research Council Centre for Medical Molecular Virology, Division of Infection and Immunity, University College London, 90 Gower Street, London WC1E 6BT, United Kingdom

## Abstract

Lentiviruses have evolved to infect and replicate in a variety of cell types *in vivo* whilst avoiding the powerful inhibitory activities of restriction factors or cell autonomous innate immune responses. In this review we offer our opinions on how HIV-1 uses a series of host proteins as cofactors for infection. We present a model that may explain how the capsid protein has a fundamental role in the early part of the viral lifecycle by utilising cyclophilin A (CypA), cleavage and polyadenylation specificity factor-6 (CPSF6), Nup358 and TNPO3 to orchestrate a coordinated process of DNA synthesis, capsid uncoating and integration targeting that evades innate responses and promotes integration into preferred areas of chromatin.

## Highlights

• HIV-1 utilises CPSF6 to suppress premature reverse transcription and target viral cores to nuclear pores.
• HIV-1 uses TNPO3 to transport the preintegration complex into the nucleus.
• HIV-1 uses this pathway to target active chromatin whilst evading innate sensors.

**Current Opinion in Virology** 2014, **4**:32–36

This review comes from a themed issue on **Virus entry**

Edited by **Mark Marsh** and **Jane A McKeating**

For a complete overview see the Issue and the Editorial

Available online 14th January 2014

1879-6257/$ – see front matter, © 2013 The Authors. Published by Elsevier B.V. All rights reserved.

**http://dx.doi.org/10.1016/j.coviro.2013.11.003**

## Introduction

Retroviruses are defined by their ability to integrate a DNA copy of their genome into the host chromatin. In order to achieve this, they must first reverse transcribe (RT) their RNA genome into double stranded DNA and then gain access to the nucleus. The majority of retrovirus families are dependent upon mitosis to access the nuclear compartment. In contrast, lentiviruses, such as human immunodeficiency virus type-1 (HIV-1), have evolved to traverse the nuclear pore complex (NPC) allowing replication in non-dividing cells such as macrophages. Whilst the molecular details of reverse transcription and integration have been well established, much remains uncertain regarding the loss of the capsid (CA) shell that protects the reverse transcription complex (RTC), a process called uncoating. In particular, the host cell cofactors on which the virus depends have been the subject of intense scrutiny of late, and as a result our understanding is rapidly progressing. It is becoming evident that the early interactions between host cofactors and the viral CA are key in determining the path taken by the viral core, and that these interactions influence downstream processes. As further details become clear we propose that this new knowledge will allow novel therapeutic interventions, and more effective use of lentiviruses as tools.

## The role of CA in early infection

### The timing of uncoating

Uncoating has been one of the most enigmatic aspects of early HIV-1 infection and is defined as the loss of the CA core from the RTC. Conflicts of size suggest that the conical core must be lost before nuclear entry: the width of the core is 50–60 nm, whereas the NPC pore diameter is ∼30 nm, but the precise timing and mechanism of uncoating remain undefined. Early biochemical analyses of cores purified from newly infected cells suggested that the HIV-1 CA core is unstable and lost soon after cell entry [1,2]. This was interpreted as CA being dispensable for subsequent viral processes. However, recent genetic data suggest that CA stays associated with the virion for longer [3^••^]. The different models for disassembly stem in part from the fact that biochemical assays measure what happens to the majority of particles, most of which do not successfully infect the cell, whereas genetic analyses can be focused on the infectious particles only, for example, by reading out infection through GFP expression [4]. Microscopy approaches have also led to conflicting conclusions, with some studies supporting cytoplasmic uncoating, whilst others have suggested later, NPC associated, uncoating [5^••^,6–8].

### A functional role for CA

Consistent with an important role for CA in the incoming phase of the life cycle, several studies have functionally linked reverse transcription and uncoating. An increasing number of studies demonstrate that suppressing reverse transcription delays uncoating, supporting a role for reverse transcription in promoting uncoating [5^••^,9,10]. Furthermore, certain CA mutants have defects in reverse transcription [11,12]. Some authors have suggested that a late role for CA, despite early uncoating, may be rationalised by partial CA uncoating in the cytoplasm. However, it is difficult to understand how partial uncoating would not lead rapidly to complete uncoating given that the CA structure appears to be unattached to its contents, and dependent on a lattice of CA-CA interactions [13]. However, it is becoming evident that mutations in CA can influence events that occur downstream of the uncoating event. HIV-1 CA has now been well established as a viral determinant for nuclear entry and the ability to infect non-dividing cells [14–16]. As discussed below, mutation of CA also impacts on the use of downstream cofactors including TNPO3, Nup358 and Nup153, all of which influence integration site targeting [3^••^,17,18^••^]. CA is therefore likely to also have consequences for expression of the viral RNA/proteins due to its influence on the provirus context within chromatin.

## Host cofactors for nuclear entry

The fact that CA impacts on viral processes even after its loss most likely results from influential interactions that occur between the CA and host cofactors before the uncoating process begins. How CA has such a central role in the behaviour of HIV-1 is gradually being uncovered by a wealth of literature surrounding the identification and role of host proteins in this stage of HIV infection. As our understanding of these data grows, so it informs our understanding of basic viral and cellular biology.

### TNPO3/nucleoporins

In 2008 genome wide siRNA screens identified a large number of putative host cofactors for HIV-1 infection [19,20]. Amongst these were the karyopherin TNPO3, also identified as an HIV-1 integrase interactor in a yeast-2-hybrid screen [21], and NPC proteins Nup153 and Nup358. Depletion of these proteins using RNA interference impaired HIV-1 infection [3^••^,17,18^••^,21–23], and also impacted on integration site selection, with integrated proviruses still being identified in genes but in regions of lower gene density [24,25^••^]. The precise step of the viral life cycle at which these cofactors act has been disputed [7,17,18^••^,21,26–31]. This has been largely due to conflicting measurements of the abundance of 2LTR circles, which are formed by components of the non-homologous end joining pathway that are found uniquely in the nucleus. Thus 2LTR circles are a much-used marker for nuclear entry. However, two recent studies elegantly demonstrated that 2LTR circle PCR assays must use primer/probe sequences that actually span the LTR-LTR junction, or they can detect the autointegrants that form as completed RT products back up at the defective NPC and integrate into each other [32^•^,33^•^]. The autointegrants in these studies were identified by sequencing the 2LTR PCR products. The use of appropriately designed primers supports a nuclear entry defect on depletion of TNPO3 [32^•^].

How might HIV-1 CA influence nuclear entry? HIV-1 CA is certainly capable of interacting with the NPC directly by binding to the cyclophilin-like domain of Nup358 [7,18^••^]. In this way Nup358 recruitment may tether a reverse transcribing virion to the NPC and orchestrate interaction with the nuclear transport machinery during the uncoating process. The isolated Nup358 Cyp domain has been shown to catalyse *cis–trans* isomerisation of the G89-P90 bond in CA using NMR techniques [34] and although it is irresistible to hypothesise that this manipulation of CA has a role in controlling uncoating, direct evidence remains elusive. Nup358 also possesses Ran binding domains, and as such could play a role in the regulation of RanGTP dependent nuclear import. TNPO3, itself a RanGTP dependent nuclear import protein, is a likely candidate for trafficking the virus towards or through the NPC. TNPO3 is capable of directly binding HIV-1 integrase (IN), but the importance of this interaction to nuclear entry has been controversial [21,22,35], not least because dependence on TNPO3 has been genetically mapped to CA [18^••^,22,25^••^,26]. However, interactions between CA and Nup358 and CPSF6 could dictate the site of uncoating, either tethered to the NPC by Nup358 or in the cytoplasm, and thus whether integrase has the opportunity to encounter TNPO3. This possibility is supported by the behaviour of HIV-1 CA mutants. For example, the CPSF6 binding mutant CA N74D infects independently of Nup358 and TNPO3 and has retargeted integration preferences. Similarly, HIV-1 CA P90A infects independently of CypA and Nup358 and also retargets integration [3^••^,18^••^]. The nuclear basket component, Nup153, has also been suggested to bind integrase, again with dependence on Nup153 being mapped to CA [17,36]. More recently, Nup153 has been shown to interact directly with CA, binding the same pocket in CA which is bound by CPSF6 [48^••^].

### CypA

It has been known for some time that cyclophilin A (CypA) plays a role in HIV-1 infection, with CypA binding to an exposed loop on the surface of the CA protein [37]. As a peptidyl-prolyl isomerase, CypA also catalyses *cis*–*trans* isomerisation of CA but, as for Nup358, an inability to separate binding and catalysis activities has hindered studies into whether isomerisation contributes to HIV-1 uncoating and infectivity [38,39]. Interaction between CA and CypA can be blocked through the use of cyclosporines or CA mutants G89V and P90A [40–42]. Despite a wealth of experimental data, understanding the role of CypA in infection has been difficult, not least because it varies between cell lines. New insight has come from the observation that blocking the interaction between CypA and CA relieves dependence on cofactors Nup358 and TNPO3, and subsequently changes integration site targeting [18^••^]. Importantly, cyclosporine (Cs) can be used to target CypA without inhibiting Nup358 Cyp recruitment. This has suggested that interaction between CypA and CA influences the course of HIV-1 infection even in circumstances where its manipulation does not reduce infectivity. The mechanism by which CypA influences the route of nuclear entry remains intriguing but poorly understood.

### CPSF6

Cleavage and polyadenylation specificity factor-6 (CPSF6) was initially described as an HIV-1 inhibitory factor when a truncation of the murine variant was identified in a cDNA screen for restriction factors [3^••^]. CPSF6 is primarily nuclear, but manipulation of the C-terminal nuclear localisation signal results in its cytoplasmic accumulation and inhibition of HIV-1 infection [3^••^,32^•^,43^••^]. Inhibition depends on direct recruitment of CPSF6 by HIV-1 CA, and the co-crystal structure of a CPSF6 derived peptide bound to CA revealed details of the interaction [44^••^]. Single point mutations in either the truncated CPSF6 or CA are sufficient to ablate binding (F321N and N74D respectively) and rescue infectivity [3^••^,28,44^••^,45]. As mentioned above, the HIV-1 CA N74D CPSF6 binding mutant has been highly informative for understanding the role of CPSF6 as an HIV-1 cofactor. This mutant integrates with retargeted integration site preferences, essentially integrating into genes randomly [18^••^]. Importantly, this mutant also becomes insensitive to depletion of Nup358 and TNPO3 [3^••^] as do other CPSF6 CA binding mutants [44^••^]. We interpret these results as showing that CPSF6 directs HIV-1 into a particular pathway of nuclear entry that requires Nup358 and TNPO3 function. CPSF6 also appears to mediate CA's control of HIV-1 reverse transcription. The interaction between C-terminally truncated CPSF6 (delta NLS CPSF6) and the viral core delays both RT and uncoating [28,32^•^]. Whilst initial reports of the capacity of truncated CPSF6 to block RT have been conflicting [3^••^,28,45], a thorough investigation has determined that these discrepancies can be mapped to CPSF6 exon structure. CPSF6 mutants with a disrupted NLS but with the natural exon structure are capable of blocking viral DNA synthesis [43^••^,46^••^]. The mechanism of this inhibition remains to be clarified, but the data support a model in which CA recruitment of CPSF6 controls reverse transcription and therefore uncoating, as well as the subsequent recruitment of host cofactors. At present, one of the most confusing observations is the fact that, as for CypA, depletion or over expression of CPSF6 does not impact HIV-1 infection or replication in cell lines [3^••^,28,43^••^]. However, given that both CypA and CPSF6 appear to influence the route of HIV-1 nuclear entry, we propose that both of these proteins are important for HIV-1 even though they do not always impact infectivity when manipulated. This hypothesis is supported by the observation that neither the Cyp binding mutant HIV-1 CA P90A, or the CPSF6 binding mutant HIV-1 CA N74D, replicate in primary human macrophages [18^••^,47] and that manipulating CypA or CPSF6 interactions in these cells causes HIV-1 to trigger innate immune DNA sensors [46^••^].

## Conclusions

**Figure 1:**
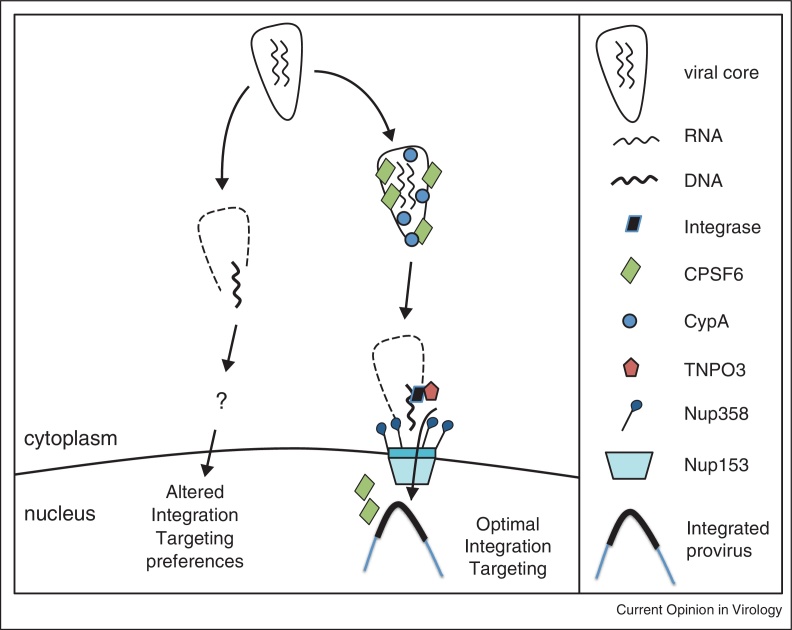
A hypothetical model of cofactor mediated HIV-1 nuclear entry and integration targeting. Shortly after entry into the cytoplasm CypA and CPSF6 are recruited to the viral core. These interactions suppress premature reverse transcription by a mechanism that remains unclear. CPSF6 recruitment allows HIV-1 to utilise the cofactors used by CPSF6 itself for nuclear entry, including TNPO3. At the NPC CA recruits the cyclophilin domain of Nup358. CPSF6 nuclear entry releases the virus enabling DNA synthesis. Nup358 use allows docking or tethering of the reverse transcription complex to the NPC, where appropriately orchestrated uncoating can expose the viral pre-integration complex for interaction with transport factors including TNPO3. In the absence of CypA or CPSF6 interaction, reverse transcription drives cytoplasmic uncoating, leading to Nup358 and TNPO3 independence and retargeted integration. We envisage a complex process of carefully orchestrated simultaneous reverse transcription, uncoating and integration events that have evolved to allow evasion of innate immune sensors. The use of proteins with a role in active transcription such as CPSF6, with its role in RNA 3′ end processing, allows HIV-1 to target transcriptionally active chromatin.

The mechanisms by which cofactors facilitate nuclear entry are still largely hypothetical and, like all good models, ours (Figure 1) is probably flawed, but testable. We note that the interpretation of data is complicated by the fact that the behaviours of NPC and nuclear transport proteins are likely to be interconnected. Thus manipulation of one, for example Nup358, has an impact on others, for example, TNPO3. It can therefore be challenging to establish whether a particular factor plays a direct role, or whether it impacts infection by regulating other members of a nuclear import pathway. However, it is reasonable to suppose that HIV-1 has evolved to make use of a connected series of proteins to optimally infect target cells and access preferred regions of the genome. We hypothesise that recruitment of CPSF6, a 3′ end mRNA processing factor, is a way to target a pathway leading to the peripheral regions of chromatin containing the active genes that HIV-1 seeks. We imagine that defining the details of such a complex situation will require the collaboration of a variety of techniques including genetic, microscopic and biochemical approaches to eventually understand the molecular mysteries of lentiviral nuclear import. We propose that cell type specificity of cofactor use is important. Whilst HIV-1 infects several different cell types *in vivo* it seems unlikely that the same cofactors will be important for infection of distantly related cells, for example, activated T cells versus terminally differentiated macrophages. Thus it will be important to remember that negative data can be difficult to interpret, particularly if they appear to be cell type specific. We are confident that eventually our greater understanding of the processes of HIV-1 reverse transcription, uncoating and nuclear transport will be fundamental to our ability to manipulate infection both therapeutically and experimentally.

## References and recommended reading

Papers of particular interest, published within the period of review, have been highlighted as:

• of special interest
•• of outstanding interest
